# Self-Healing, Solvent-Free, Anti-Corrosion Coating Based on Skin-like Polyurethane/Carbon Nanotubes Composites with Real-Time Damage Monitoring

**DOI:** 10.3390/nano13010124

**Published:** 2022-12-26

**Authors:** Hui Kong, Xiaomin Luo, Peng Zhang, Jianyan Feng, Pengni Li, Wenjie Hu, Xuechuan Wang, Xinhua Liu

**Affiliations:** 1 College of Bioresources Chemical and Materials Engineering, Shaanxi University of Science & Technology, WeiYang District, Xi’an 710021, China; 2 National Demonstration Center for Experimental Light Chemistry Engineering Education, Shaanxi University of Science & Technology, WeiYang District, Xi’an 710021, China; 3 Tongxiang Affairs Center of Quality and Technical Supervision, Tongxiang 314599, China; 4 National Wool Knitwear Quality Supervision Inspection Center (Zhe Jiang), Tongxiang 314599, China

**Keywords:** solvent-free polyurethane, carbon nanotubes, anti-corrosion, self-healing, damage monitoring

## Abstract

Self-healing anti-corrosion materials are widely regarded as a promising long-term corrosion protection strategy, and this is even more significant if the damage can be monitored in real-time and consequently repaired. Inspired by the hierarchical structure of human skin, self-healing, solvent-free polyurethane/carbon nanotubes composites (SFPUHE-HTF-CNTs) with a skin-like bilayer structure were constructed. The SFPUHE-HTF-CNTs were composed of two layers, namely, a hydrophobic solvent-free polyurethane (SFPUHE-HTF) containing disulfide bonds and fluorinated polysiloxane chain segments consisting of a self-healing layer and CNTs with good electrical conductivity consisting of a corrosion protection layer, which also allowed for the real-time monitoring of damage. The results of corrosion protection experiments indicated that the SFPUHE-HTF-CNTs had a low corrosion current density (8.94 × 10^−9^ A·cm^−2^), a positive corrosion potential (−0.38 V), and a high impedance modulus (|Z| = 4.79 × 10^5^ Ω·cm^2^). The impedance modulus could still reach 4.54 × 10^4^ Ω·cm^2^ after self-healing, showing excellent self-healing properties for anti-corrosion protection. Synchronously, the SFPUHE-HTF-CNTs exhibited a satisfactory damage sensing performance, enabling the real-time monitoring of fractures at different sizes. This work realized the effective combination of self-healing with corrosion protection and damage detection functions through a bionic design, and revealed the green, and low-cost preparation of advanced composites, which have the advantage of scale production.

## 1. Introduction

Metal corrosion is endangering various industries and compared with earthquakes, tsunamis, and other natural disasters, metal corrosion is silent but equally destructive, causing substantial economic losses every year [1]. According to the statistics, the financial losses caused by metal corrosion are about six times that of natural disasters [2]. Moreover, the safety risks for facilities and equipment caused by metal corrosion may include the threat to people’s lives; therefore, the corrosion protection of metals has been a crucial problem that needs to be solved in the scientific and engineering community. Applying organic coatings to metal surfaces is an effective strategy to achieve corrosion protection [3,4,5]. However, a vast majority of anti-corrosive coatings are susceptible to being damaged by external mechanical forces and producing micro-cracks during the application process, resulting in a severe deterioration in their anti-corrosive performance [6]. At present, the coating damages are mostly repaired manually, which not only increases the labor cost, but also may be restricted by the construction conditions, and restoring the coating surface to its original state after repair is not easy. Therefore, the development of self-healing anti-corrosion coatings with a real-time damage monitoring function can detect micro-cracks and repair them in time, which in turn can extend the service life of the coatings, reduce the maintenance costs, and protect the safety of the substrates.

Currently, there are two common methods for preparing self-healing anti-corrosion coatings. The first involves adding microcapsules loaded with healing agents or corrosion inhibitors to the polymer matrix [7]. The loadings inside the microcapsules are released to the defects when the coating is destroyed. Under certain circumstances, this forms a barrier film over an exposed substrate, protecting it from the corrosive media. Fu et al. [8] compounded polycaprolactone (PCL) nanofibers with 2-mercaptobenzothiazole-loaded halloysite nanotubes (HNTs-MBT) and deposited them on the surface of a metal substrate to form an interconnected fiber network. The encapsulated MBTs could be released by pH-triggering and repair scratches in a thermal-initiated manner, showing a great potential for corrosion protection applications. However, compared to highly cross-linked polymers, microcapsules are not only fragile and vulnerable, but also have a limited internal corrosion inhibitor loading, making it challenging to achieve long-lasting corrosion protection [9]; therefore, the second approach, based on the dynamic bonding of polymer resins, has become more commonly used [10]. With this method, broken molecular chains in the damaged coating are reconnected by dynamic interactions to re-establish a physical barrier to protect the metal substrate [11]. This shows an excellent cycling performance as no external repair agent is required. The commonly used dynamic chemical bonds for self-healing are hydrogen bonds [12,13], DA bonds [14,15,16], disulfide bonds [11,17,18], etc. Among them, disulfide bonds are of great interest to researchers because they can be activated at moderate temperatures (60–90 °C) or under UV irradiations [19,20]. Liu et al. [21] fabricated a novel self-healing composite coating on a magnesium alloy based on the dual effects of a corrosion inhibitor, namely, M-16, embedded in a micro-arc oxidation (MAO) coating and a self-healing polyurethane modified by disulfide bonding. The inhibitor M-16 released from the porous MAO coating improved the corrosion resistance of the damaged layer and the physical damage on the coating could be repaired by a heat treatment; however, studies on disulfide-bonded, self-healing, anti-corrosion coatings are mostly solvent-based. With the successive introduction of environmental protection policies in various countries, the need to replace solvent-based coatings with solvent-free coatings is inevitable.

More significantly, self-healing anticorrosive coatings also face a pivotal problem that is often overlooked; that is, although self-healing can reduce the maintenance costs and improve the service life, usually in the early stages of product damage, the resulting micro-crack is tiny and hardly detectable. To avoid a micro-crack from growing into a fatal defect, there is an urgent need to develop smart, anticorrosive coatings with damage monitoring capabilities [22]. Several studies on damage monitoring materials have been reported. For example, Wei et al. [23] developed a MWCNTs/CB waterborne conductive smart coating and the damage to composite laminates with various thicknesses at different impact loads was analyzed. Furthermore, a damage localization prototype was proposed to determine the damage location. Qureshi et al. [24] prepared a nylon/silver strain sensor wire, and it showed a good reproducibility in an electrical response signal when subjected to the same tensile load. The as-designed strain sensor wire not only detected changes in the mechanical behavior and strain deformation during material elongation, but it monitored and identified the type of damage in real-time. Little has been reported, however, on the damage detection of crack sizes, which is the key to achieving damage monitoring.

Polyurethane exhibits outstanding flexibility, good mechanical strength and chemical resistance stability [16,25]. Solvent-free polyurethane is a new green and environmentally friendly coating material that has emerged recently and can replace solvent-based polyurethane. Disulfide bonds (S–S) formed by symmetrical exchange reactions involve only changes between two atoms [26]. The dynamic covalent crosslinking systems constructed using them all have superior photo- and thermal-restoration effects. Fluorinated polysiloxanes (HTF) can impart hydrophobicity to materials while forming hydrogen bonds with polar groups to facilitate the self-healing process. In light of these considerations, a self-repairing, solvent-free polyurethane containing dynamically covalently bonded disulfides (HEDS) and HTF was synthesized, abbreviated as SFPUHE-HTF. In nature, the hard outer layer covering the soft matrix is a common strategy to resist abrasion or penetration, as in human skin, where the harder epidermis acts as a barrier and defense layer to prevent microbial invasion of the softer dermis and to cushion against external mechanical damage. Inspired by the layered structure of human skin, a solvent-free polyurethane/carbon nanotubes composite (SFPUHE-HTF-CNT) with a skin-like bilayer structure was constructed by spraying a CNTs/waterborne polyurethane (WPU) dispersion on the surface of a SFPUHE-HTF coating as the anti-corrosion protection layer through a simple, low-cost and rapid spraying technique. There were benefits from the conductive network formed by the CNTs in the protective layer, with the coating having an excellent electrical conductivity. When the coating was damaged, its conductivity would change, and the damage could be monitored in real-time. The preparation method has the advantages of being green, environmentally-friendly, highly efficient and conducive to large-scale production, which provides a new strategy for developing innovative anti-corrosion coatings.

## 2. Experimental Methods

### 2.1. Materials

Polyethylene adipate diol (CMA-1000), polytetrahydrofuran ether diol (PTMG-1000), and isophorone diisocyanate (IPDI) were procured from Yantai Daocheng Chemical Co., Ltd. (Yantai City, China), and polyol was used after vacuum drying at 110 °C for 4 h. 2-hydroxyethyl diisocyanate Sulfide (HEDS) was obtained from Shanghai McLean Biochemical Technology Co., Ltd. (Shanghai, China) Dibutyltin dilaurate (DBTDL) was purchased from Shanghai Aladdin Biotechnology Co., Ltd. (Shanghai, China) 1,4-butanediol (BDO) was supplied by the Tianjin Fuchen Chemical Reagent Factory (Tianjin, China). Hydroxyl-terminated fluorine-containing polysiloxane (HTF) was produced by Shanghai Silicon Mountain Polymer Materials Co., Ltd. (Shanghai, China) A CNTs aqueous dispersion (TNWDM-M2) was provided by Chengdu Organic Chemistry Co., Ltd. (Chengdu, China), the Chinese Academy of Sciences, and WPU (XWB3030) was purchased from Asahikawa Chemical (Suzhou) Co., Ltd (Suzhou, China).

### 2.2. Preparation of SFPUHE-HTF and SFPUHE-HTF-CNT Coatings

First, CMA-1000 (11 mmol, Mn = 1000), PTMG-1000 (11 mmol, Mn = 1000), IPDI (44 mmol, Mn = 222.32), and DBTDL (2.0 μL) were sequentially added to a three-neck flask, and the mixture was heated to 80 °C and reacted for 3 h to obtain the prepolymer (Pr-PU). Then, HEDS (60 mmol, Mn = 154.25) was employed to the reaction system to expand the chain for 30 min, and the mixture was taken out and evenly coated on the release paper (with a coating thickness of 60 μm). Considering that the molding of solvent-free polyurethane involves a chain growth and cross-linking reaction, direct high-temperature heating tends to lead to a too-fast cross-linking speed, which then leads to an uneven film formation. Consequently, a staged heating process was implemented: the mixture was (i) heated at 60 °C for 1 h; (ii) heated to 90 °C for 2 h; (iii) heated to 120 °C for 4 h; then (iv) lowered to 70 °C for 8 h, and then taken out to obtain the disulfide-bonded, self-healing, solvent-free polyurethane (SFPU-HE). The SFPU-HE0 was prepared by replacing the HEDS with BDO as a control group. Meanwhile, 12% of the Pr-PU mass of HTF was added to replace part of the HEDS, and the reaction was stirred for 20 min at 80 °C to obtain a self-repairing, solvent-free polyurethane containing HEDS and HTF (SFPUHE-HTF), then scraped onto the release paper and placed in the oven at 60 °C for 1 h for cross-linking. Finally, the CNTs aqueous dispersion was mixed with an appropriate amount of polyurethane emulsions and ultrasonically dispersed for 30 min, and sprayed evenly onto the incompletely-solidified SFPUHE-HTF coating to improve the adhesion between the CNTs layer and the polyurethane layer. Then, the obtained material was dried at 80 °C for 30 min, cycling the spraying/drying process several times to prepare a composite coating with different CNT contents and abbreviated as SFPUHE-HTF-CNTs-x, where x denotes the weight content of the CNTs (i.e., x = 1, 2, 3, 4).

### 2.3. Characterization

A Fourier-transform infrared (FTIR) spectrophotometer (Vertex 70, Bruker, Bremen, Germany) was applied to record the FTIR spectra at the wavenumbers of 4000~400 cm^−1^. The Raman spectra were obtained using a Raman spectrometer (inVia, Renishaw, Gloucestershire, UK) with 514 nm of laser excitation. An XRD was performed using an X-ray diffractometer (D/max2200PC, Bruker, Bremen, Germany) at a tube pressure of 40 kV, a scanning range of 2θ from 10°~60° and a scanning speed of 5°/min. A TGA was carried out on a thermogravimetric analyzer (Q500, TA Company, New Castle, DE, USA) over the temperature range from 30 °C to 800 °C at a heating rate of 5 °C/min under a nitrogen atmosphere. A DSC was performed on a DSC-Q2000 instrument (TA Company, New Castle, DE, USA) using a heating rate of 10 °C/min and purging nitrogen at a flow rate of about 50 mL/min. The self-healing process of the specimens was investigated under a polarized optical microscope (POM) equipped with a programmed warming heating station to observe the cracks (made by blades). A KH-8700 ultra-depth-of-field 3D microscope (Japan HIROX Corporation, Tokyo, Japan) was used to observe the self-healing process of the samples in three dimensions. To test the UV self-healing performance of the SFPUHE-HTF-CNTs, a film with a length, width and height of 3 cm × 2 cm × 0.4 cm, respectively, was cut with a sharp knife, and then laid on a PTFE sheet and placed in a UV chamber. The UV irradiation was performed at an energy value of 200 and the repair of the fracture surface was observed at regular intervals. According to the GB/T 528-1998 standard, the SFPUHE-HTF-CNT film was cut into a dumbbell shape (40 mm × 10 mm × 0.8~1.0 mm) and tested for tensile strength using a universal tensile machine (AI-7000 NGD, Gotech, Dongguan, China) at a rate of 100 mm/min, followed by cutting out a constant width crack with a blade and then testing the tensile strength of the sample after repairing, using Equation (1) to characterize the self-repair efficiency of the polyurethane samples. The water contact angle of the material surface was measured using a Dataphysics video optical contact angle measuring instrument, with the volume of water droplets set at 5 μL, while the average value was taken at five random points on the membrane.

(1) $$\mathrm{Healing}\;\mathrm{effecieny}\left(\%\right)=\frac{\mathrm{Tensile}\;\mathrm{strength}\left(n\right)\mathrm{Cycle}}{\mathrm{Tensile}\;\mathrm{strength}\;\mathrm{Original}}\times 100\%$$

To characterize the surface morphology of the SFPUHE-HTF-CNTs, a scanning electron microscope (FEI Verios) was applied at different magnifications of the coating surface at the accelerating voltage of 20 kV. The samples for the SEM observations were coated with gold on the surface. The self-healing properties of the SFPUHE-HTF-CNT films were tested by an electron microscope (Suolan, G1600). The electrochemical corrosion behavior in a NaCl solution (3.5 wt%) was measured by an electrochemical workstation (PARSTAT4000, Princeton, NJ, USA). In addition, the optical microscope was used to observe the corrosion on the surface of the bare and coated steel sheet immersed in a NaCl solution (3.5 wt%, 1 h, 24 h, 7 d, 14 d) before and after the coating was damaged (made of blades) at a magnification of 1000. The surface chemical compositions of the SFPUHE-HTF-CNTs before and after self-healing were carefully detected by energy-dispersive spectroscopy (EDS, Tescan, MIRA3). A SZT-2B four-probe tester (Suzhou Tongchuang Electronics Co., Ltd., Suzhou, China) was used to measure the electrical conductivity of the SFPUHE-HTF-CNTs coating before and after the damage. The sensing performance of the SFPUHE-HTF-CNTs coating was analyzed by an I-t curve test in a PARSTAT4000 electrochemical workstation.

## 3. Results and Discussion

### 3.1. Fabrication and Structural Characterization of SFPUHE-HTF and SFPUHE-HTF-CNTs

**Figure 1:**
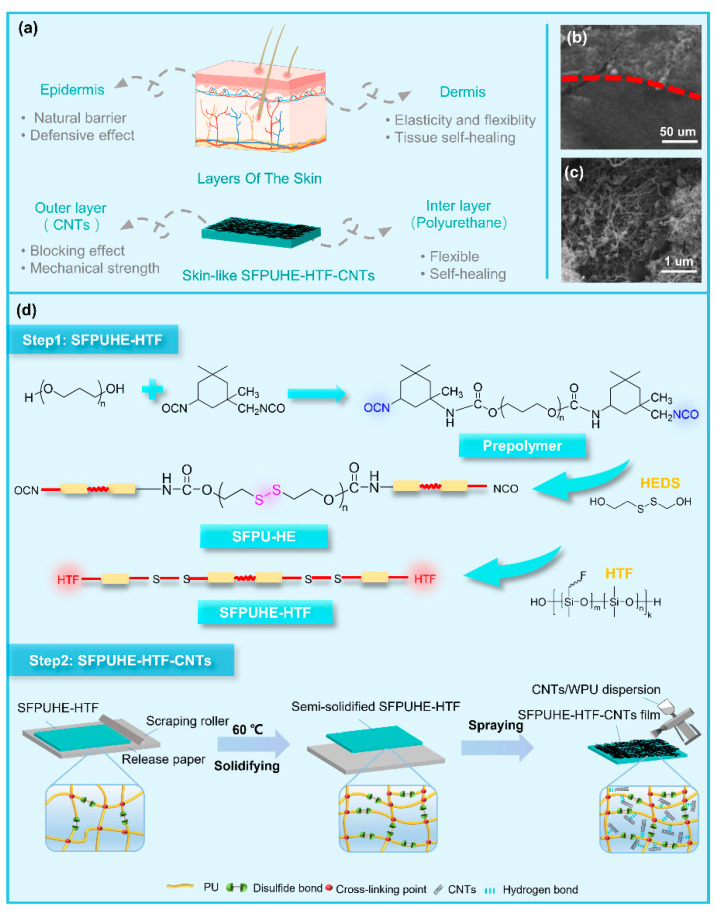
(**a**) Schematic diagram of the design concept for preparing the skin-inspired, bilayer solvent-free polyurethane/carbon nanotubes composites (SFPUHE-HTF-CNTs). The SEM images of (**b**) the bilayer structure and (**c**) the outer CNTs layer. (**d**) The fabrication process of SFPUHE-HTF-CNTs.

Inspired by the multi-layered structure of human skin, the bilayer skin-like coating of SFPUHE-HTF-CNTs was constructed by the scraping and spraying techniques in this paper. As shown in Figure 1a, the design of the skin-like coating was based on two principles. First, the similarity of the material properties and structure. The skin consists of a thinner, harder epidermal layer and a thicker, softer dermal layer [27]. Correspondingly, by controlling the thickness of scraping and the amount of CNTs sprayed, the skin-like bilayer coating designed as demonstrated here consisted of a thinner rigid CNT layer and a thicker flexible polyurethane layer. Secondly, a similarity of the functions, since the epidermis layer of skin provides a barrier for the substrate, and the dermis layer confers good flexibility and elasticity to the skin, providing insulation, a buffering external shock and a self-healing function of skin tissue damage [28]. Similarly, the polyurethane layer composed of flexible molecular chains containing dynamic hydrogen and disulfide bonds provided flexibility, elasticity and self-healing functions to the coating, while the CNT layer was relatively rigid and dense, which endowed the polyurethane elastomer layer with a significant barrier effect and mechanical strength [29]. Figure 1b shows that the SFPUHE-HTF-CNTs had a distinct layered structure, with the inner layer composed of self-healing polyurethane and the outer layer composed of CNTs. Additionally, the outer layer was enlarged as shown in Figure 1c, from which it can be seen that the CNTs were densely tangled and, thus, laid excellent foundations for the subsequent shielding protection. The preparation process of the skin-like SFPUHE-HTF-CNTs coating is shown in Figure 1d, and no organic solvent was used in the preparation process, which was a green and clean production approach.

**Figure 2:**
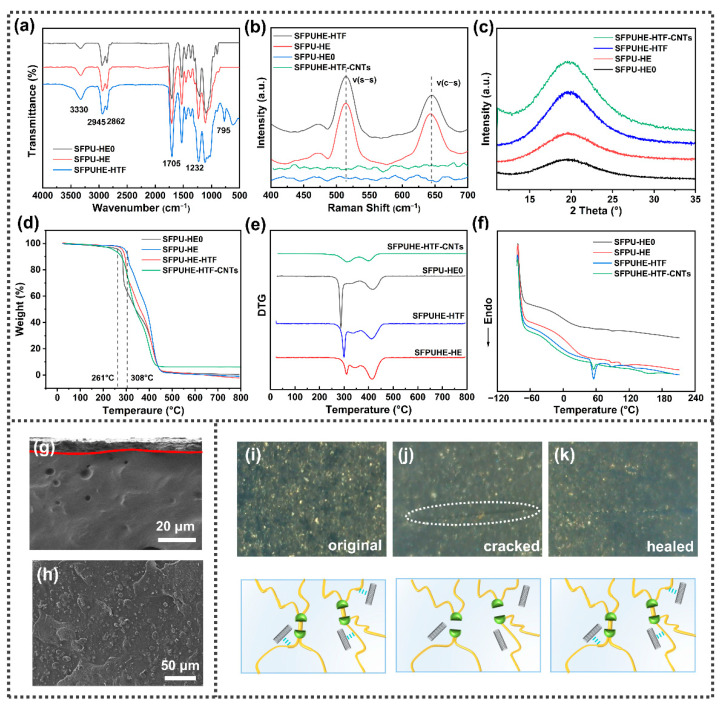
Structural characterization of self-healing polyurethanes: (**a**) FTIR spectra; (**b**) Raman spectra; (**c**) X-ray diffraction patterns; (**d**) thermogravimetric curves; (**e**) micro-quotient thermogravimetric curves; (**f**) DSC curves. The SEM images of the (**g**) cross section (500×) and (**h**) surface (1000×) morphology of SFPUHE-HTF-CNTs. (**i**–**k**) Self-healing process of SFPUHE-HTF-CNTs (photographed by microscope); the inset shows a schematic illustration of the healing mechanism.

The polyurethane inner layer imparts flexibility and self-healing properties to SFPUHE-HTF-CNTs, and whether it is successfully synthesized will affect the overall performance of the material. Therefore, the structure of the self-healing polyurethanes was firstly characterized, and their infrared spectra are shown in Figure 2a. The absorption bands at 3332 cm^−1^, 3331 cm^−1^, 3328 cm^−1^ and 3317 cm^−1^ corresponded to the stretching vibrations of the N-H bond in the SFPU-HE0, SFPU-HE, SFPUHE-HTF and SFPUHE-HTF-CNTs, respectively. It was evident that the N–H absorption bands shifted to lower wavenumbers, indicating an increase in the degree of hydrogen bonding with the addition of HEDS, HTF and CNTs. Moreover, the absorption bands at 2945 cm^−1^ and 2862 cm^−1^ were attributed to the stretching vibration of –CH_3_ and –CH_2_– bonds in the self-healing polyurethanes. The absorption bands appeared at 1705 cm^−1^ and 1232 cm^−1^ belonging to C=O and C–O–C bonds, respectively. As shown in the spectrum of the SFPUHE-HTF and SFPUHE-HTF-CNTs, the appearance of a new minor peak at 795 cm^−1^ was assigned to Si–CH_3_. The appearance of all these above absorption bands proves the successful preparation of self-healing polyurethanes. Moreover, there were no new absorption bands in the spectrum of SFPUHE-HTF-CNTs, indicating that there was only a physical mixing between the CNTs and polyurethane without chemical bonding interactions. Figure 2b illustrates the Raman spectrum of the self-healing, disulfide-bonded polyurethanes. Both the SFPU-HE and SFPUHE-HTF showed the corresponding absorption bands of S–S and C–S at 512 cm^−1^ and 640 cm^−1^, respectively. Furthermore, it was found that after some of the disulfide bonds were replaced by HTF, the intensity of the bands corresponding to disulfide bonds in the SFPUHE-HTF spectrum decreased relative to that of the SFPU-HE, indicating that the disulfide bond had been successfully cross-linked to the polyurethane molecular chain. In addition, it is worth mentioning that since the detection depth of the Raman laser was only about 10 nm, the SFPUHE-HTF-CNTs did not show the characteristic Raman shifts of sulfur-containing bonds, but showed the D and G peaks corresponding to CNTs at 1353 cm^−1^ and 1583 cm^−1^ (Figure S1), further proving the successful preparation of the SFPUHE-HTF-CNTs. To explore the crystallinity of polyurethane, an XRD was implemented, and the results are presented in Figure 2c. For the SFPU-HE0, SFPU-HE, SFPUHE-HTF and SFPUHE-HTF-CNTs, wide diffraction reflexes appeared at 2θ = 20°, representing the low crystallinity of the self-healing polyurethanes, which can be attributed to the fact that in polyurethane macromolecules, hard segments dominated by isocyanates and chain expanders and soft segments dominated by polyols are thermodynamically incompatible, and a microphase separation state will occur, resulting in the poor crystallinity of polyurethane [30,31].

TGA was performed to evaluate the thermal stability of the self-healing polyurethanes, as depicted in Figure 2d, and the initial decomposition temperature of the SFPU-HE0 increased from 261 °C to 308 °C of SFPU-HE. This phenomenon was ascribed to the increase of the content of the hard segments in the SFPU-HE by the introduction of disulfide bonds, leading to a higher cohesion energy and eventually to a higher thermal stability. However, the initial decomposition temperature of the SFPUHE-HTF was reduced to 295 °C, which was due to the lower thermal stability of the HTF with a decomposition temperature of around 230 °C, and its introduction lowered the overall decomposition temperature of the SFPUHE-HTF. Moreover, the SFPUHE-HTF-CNTs had the lowest initial decomposition temperature (245 °C), which may have been due to the low heat resistance of the WPU binder added during the preparation process; however, there was still a 6.09% weight residue at the end of the decomposition, which proves that the overall heat resistance of the composites had been improved after the addition of the CNTs. The micro-quotient thermogravimetric curve (DTG) of the self-healing polyurethanes is displayed in Figure 2e. All the samples exhibited two inflection points, indicating two decomposition process stages [32]. As for the SFPUHE-HTF and SFPUHE-HTF-CNTs, the first inflection point occurred at 298 °C and 315 °C, respectively. This could be attributed to the decomposition of carbon chains in the polyol soft segment. The second inflection point could be observed when the temperature reached 413 °C and 406 °C, respectively, which was attributed to the decomposition of some functional groups on the hard segment, such as the –NHCOO– bonds. It is worth noting that the weight loss temperature for both the two samples was higher than 286 °C when compared with the SFPU-HE0, indicating that the introduction of disulfide bonds and HTF and CNTs had improved the thermal stability of the SFPUHE-HTF. In addition, the SFPUHE-HTF-CNTs showed a smoother peak at 315 °C when compared with the other three samples, indicating that the SFPUHE-HTF-CNTs had a lower weight loss rate at 315 °C, further suggesting that the addition of CNTs can effectively slow down the thermal decomposition of the soft segment. To further investigate the thermal reversibility of polyurethanes with disulfide bonds and HTF in the main chain, the variations of SFPU-HE0, SFPU-HE, SFPUHE-HTF and SFPUHE-HTF-CNTs under the effect of temperature were tested by DSC. As illustrated in Figure 2f, all four curves displayed two steps, representing the glass transition temperatures of the soft and hard polyurethane segments, respectively. The glass transition temperature of the soft segment determines the thermal properties of polyurethane. Through the “isometric method”, the glass transition temperatures of the SFPU-HE0, SFPU-HE, SFPUHE-HTF and SFPUHE-HTF-CNTs were determined as 0 °C, 0 °C, −17.7 °C and−16.9 °C, respectively (Figure S2a). This proves that the glass transition temperatures of the soft segments of the self-healing polyurethanes were lower than room temperature, indicating that the polyurethanes were highly elastic at room temperature. Their chain segments were highly mobile, which was conducive to the dynamic reversible disulfide bond translocation exchange reaction in the molecular chain; thus, facilitating the self-healing process. Additionally, the SFPUHE-HTF and SFPUHE-HTF-CNTs presented a prominent heat absorption peak near 60 °C, which was attributed to the exchange reaction of disulfide bonds affected by temperature, thus, reassembling the original broken bonds into new disulfide bonds, and causing the system to absorb heat [33]. More importantly, the appearance of this heat absorption peak was a robust and powerful basis for the temperature selection of further self-healing.

The CNT outer layer provided protection and functionality for the SFPUHE-HTF-CNTs, and the preparation of dense and homogeneous CNT layers helped to maximize their functionality. It can be seen from the SEM cross-section in Figure 2g that the SFPUHE-HTF-CNTs had an obvious bi-layer structure. During the spraying process, the dispersion of CNTs in the polymer matrix is the key issue [34]. As shown by the SEM surface images of the SFPUHE-HTF-CNTs composites (Figure 2h), CNTs were uniformly dispersed in the polymer matrix without apparent aggregates, which was conducive to the formation of perfect and effective barrier channels and conductive pathways, thus, improving the anti-corrosion and sensing performances of the SFPUHE-HTF-CNTs coatings. Figure 2i–k shows the physical pictures of the SFPUHE-HTF-CNTs before and after the self-healing and the schematic diagram of the healing mechanism. The surface of the original SFPUHE-HTF-CNTs showed a uniform black color, and a bright crack appeared as in Figure 2i after breaking. When heated at 60 °C for 16 min, the scratches on the surface of the coating almost disappeared and only slight traces of healing residue could be observed (Figure 2k). Notably, when human skin is injured, blood clots on the wound and gradually self-heals. This process is dominated by the upward diffusion of active keratinocytes [35], and similar to this, the dynamic chemical bonding promoted self-healing of the coating. The self-healing of the SFPUHE-HTF-CNTs was due to the translocation exchange reaction of disulfide bonds on the one hand, and HTF forming dynamic hydrogen bonds with the polar groups in the polyurethane chain segments on the other hand. The two synergistically drove the repair of the upper CNTs, thus, facilitating the self-healing process, which provided favorable conditions for the subsequent realization of long-lasting corrosion and damage-sensing properties that can be used for self-healing.

### 3.2. Self-Repair Performance and Repair Mechanism of SFPUHE-HTF and SFPUHE-HTF-CNTs

**Figure 3:**
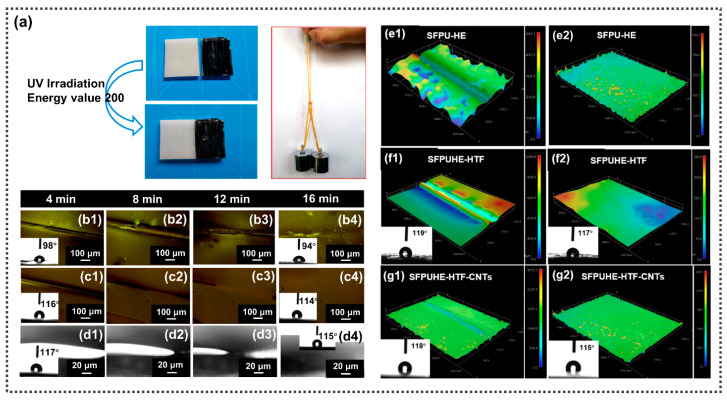
(**a**) Comparison of SFPUHE-HTF-CNTs before and after healing under UV light irradiation at the energy of 200; the repaired sample was not pulled off under two 100 g weights. (**b**) Images of the self-healing process of the sample taken by POM during the same time interval at 60 °C. (**b1**–**b4**) Disulfide-bonded self-healing polyurethane (SFPU-HE); (**c1**–**c4**) disulfide-bonded and fluorinated polysiloxane containing self-healing polyurethane (SFPUHE-HTF); (**d1**–**d4**) SFPUHE-HTF-CNTs; the inset diagrams show the contact angles of SFPUHE-HTF and SFPUHE-HTF-CNTs before and after repair, respectively. The 3D images (200×) of the surface morphology of the sample before and after repair photographed by super depth-of-field microscopy. (**e1**,**e2**) SFPU-HE; (**f1**,**f2**) SFPUHE-HTF; (**g1**,**g2**) SFPUHE-HTF-CNTs; the inset diagrams of f1, f2 and g1, g2 show the 363 contact angles of the SFPUHE-HTF and SFPUHE-HTF-CNTs before and after repair at 60 °C.

The self-healing properties of the SFPUHE-HTF and SFPUHE-HTF-CNTs were systematically investigated by UV irradiation, polarized light microscopy and ultra-deep field microscopy. The self-healing process of the SFPUHE-HTF-CNTs under UV irradiation is illustrated in Figure 3a. Firstly, half of the sample was stained and then cut along a partition line and placed in the UV chamber at an energy value of 200 for 48 h. From Figure 3a, it can be seen that the two parts of the section were fused into one. The weight-bearing capacity of the repaired section was tested using two 100 g weights and it was not pulled off, indicating that the SFPUHE-HTF-CNTs could self-repair under UV light. Significantly, a light-responsive self-healing coating has the advantages of delivering an eco-friendly, rapid, remote and precise repair of damaged areas, which is more convenient for coating repair outdoors [36].

According to the DSC curve in Figure 2f, the SFPUHE-HTF and SFPUHE-HTF-CNTs showed a distinct heat absorption peak at 60 °C; therefore, the self-healing behaviors of the SFPU-HE, SFPUHE-HTF and SFPUHE-HTF-CNTs were observed at 60 °C under a polarized light microscope equipped with a heating stage. All three samples were cut with a sharp knife in the same size (i.e., 1 cm long, and 20 μm wide). Figure 3(b1–b4) shows that the surface cut of the SFPU-HE gradually disappeared and was utterly repaired after 16 min at 60 °C, but that some bubble structures appeared, and the water contact angle changed from 98° before the cut to 94° after the repair. As displayed in Figure 3(c1–c4), the cut marks on the surface of the SFPUHE-HTF disappeared in the same time-frame and no bubbles appeared on the surface, probably due to the leveling effect of the low surface energy HTF. Notably, the hydrophobicity also showed a favorable recoverability, with a static water contact angle of 116° before cutting to 114° after the repair at the damaged location. Similarly, the cut marks disappeared after the SFPUHE-HTF-CNTs were repaired at 60 °C for 16 min, and the sample surface remained flat and smooth. The water contact angle changed from 117° before the cut to 115° after the repair (Figure 3(d1–d4)). It was seen that all the samples could achieve self-healing at 60 °C for 16 min, which would be favored for further application in self-healing, anti-corrosion coatings.

The morphologies of the SFPU-HE, SFPUHE-HTF and SFPUHE-HTF-CNTs before and after self-repair were analyzed by super-field microscopy. The e1, e2, f1, f2 and g1, g2 in Figure 3 correspond to the morphologies of the SFPU-HE, SFPUHE-HTF and SFPUHE-HTF-CNTs before and after self-healing, respectively, where the f1, f2 and g1, g2 are em-bedded with contact angle test plots. As shown in Figure 3(e1,f1,g1), the cracks were obvious before the repair, and the results are shown in Figure 3(e2,f2,g2) after 16 min of repair at 60 °C. The contact angles of the f1 and f2 at the same position changed from 119° before cutting to 117° after the repair; the contact angles of the g1 and g2 at the same place changed from 118° to 115°, and all the specimens showed a relatively flat state at the healed area. These results indicate that all the samples had favorable self-healing properties, which were consistent with the findings revealed by the UV irradiation and polarized light microscopy. Moreover, the coating had a mild thermally-stimulated responsive self-healing condition, thus avoiding the risk of material degradation due to localized concentrated heating. The environmental friendliness and precision of light-stimulated, responsive self-healing allows for contactless repair over a long distance, thus, being promising for a broader range of applications.

**Figure 4:**
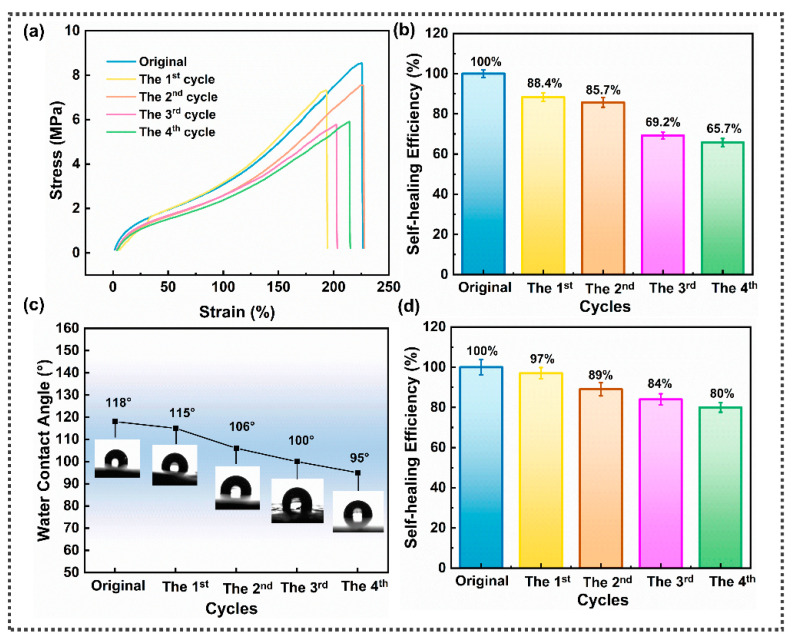
(**a**) Stress-strain curves of the original and repaired SFPUHE-HTF-CNTs; (**b**) the mechanical repair efficiency of SFPUHE-HTF-CNTs; (**c**) static water contact angles of the original and repaired SFPUHE-HTF-CNTs; (**d**) the hydrophobic self-healing efficiency of SFPUHE-HTF-CNTs.

To quantify the self-repair performance of the SFPUHE-HTF-CNTs, tensile tests were carried out to compare the tensile strength of the healed samples and the original (uncut) sample, with the self-healing efficiency calculated by Equation (1) [37]. As presented in Figure 4a, the maximum stress of the SFPUHE-HTF-CNTs gradually decreased with the increase in the number of cycles, and the repair efficiency of the 1st cycle and the 4th cycle was 88.4% and 65.7%, respectively (Figure 4b). A static contact angle tester was used to measure the water contact angle to evaluate the hydrophobicity of the SFPUHE-HTF-CNTs, and the results are shown in Figure 4c,d. The static water contact angle ranged from the original 118° to the fourth repaired 95°, and all cycles showed good hydrophobicity, with a repair efficiency of 97% in the first cycle and 80% in the fourth cycle.

**Figure 5:**
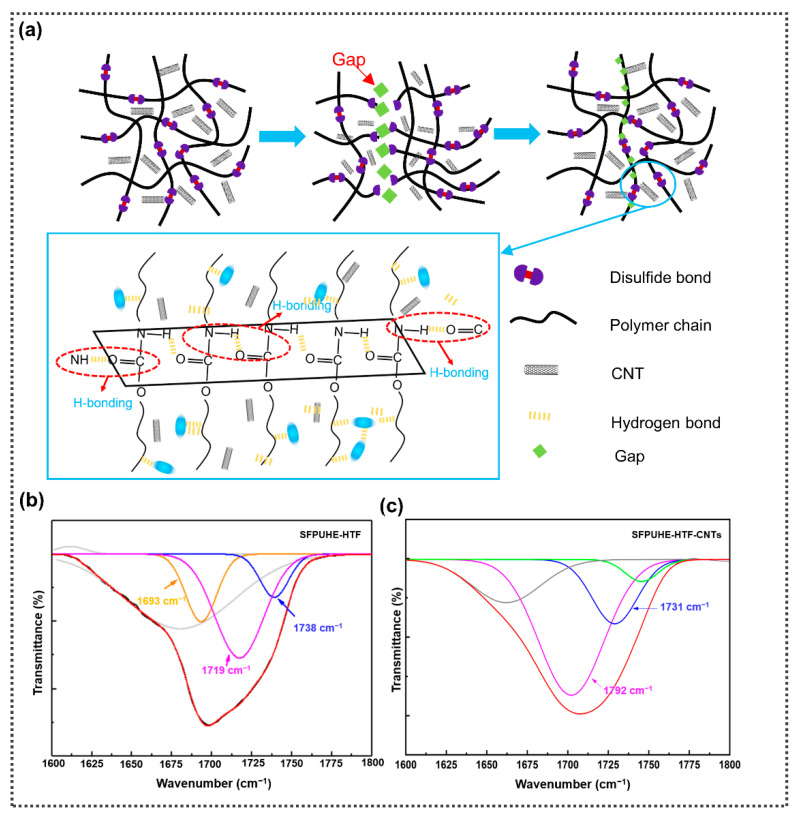
(**a**) Schematic diagram of the self-healing mechanism of SFPUHE-HTF-CNTs; (**b**) the FT-IR Gaussian split-peak plots of SFPUHE-HTF; (**c**) the FT-IR Gaussian split-peak plots of SFPUHE-HTF-CNTs.

The self-healing mechanism of the SFPUHE-HTF-CNTs is shown in Figure 5a. On the one hand, the broken disulfide bond could be reconnected with the other one to generate a new disulfide bond, i.e., the translocation exchange reaction [38]. On the other hand, an introduction of HTF replaces part of the disulfide bond, and although the content of a disulfide bond is reduced, the introduction of HTF increases the degree of hydrogen bonding of polyurethane, studies have shown that the existence of dynamic hydrogen bonding will promote the healing process of polyurethane [39,40,41]. Figure 5b shows the properties of the FT-IR spectral bands of the vibrations of the C=O group in the SFPUHE-HTF. The vibrational region of C=O was obtained by Gaussian splitting into three peaks: the stretching vibrational region of the C=O group with free hydrogen bonding appeared at 1738 cm^−1^, the C=O group with ordered hydrogen bonding at 1693 cm^−1^, and the C=O group with disordered hydrogen bonding at 1719 cm^−1^, in general agreement with the literature [42,43]. In addition, Figure 5c shows the FT-IR Gaussian split-band plots of the SFPUHE-HTF-CNTs. The bands of the vibrations of the C=O group in the SFPUHE-HTF-CNTs mainly obtained two bands through Gaussian splitting: the stretching vibration region of the C=O group with hydrogen bonding appeared at 1702 cm^−1^, and the free C=O group appeared at 1731 cm^−1^, basically consistent with the literature [44]. Generally, the peak area values of a free C=O group and a hydrogen bonded C=O group can be obtained through peak fitting. According to the fitting data, the area representing the hydrogen bonded C=O group in the SFPUHE-HTF was 43.15%, representing a high degree of hydrogen bonding. It is noteworthy that the degree of hydrogen bonding was increased when CNTs were added, and the area of the C=O group representing hydrogen bonding in the SFPUHE-HTF-CNTs was as high as 57.46%, which was 1.2% higher compared to the SFPUHE-HTF. This may have been because the CNT dispersion contained some hydroxyl groups, which could form hydrogen bonds with the polar groups in the polyurethane when added to the polyurethane, thus, promoting the self-healing of the composite.

**Figure 6:**
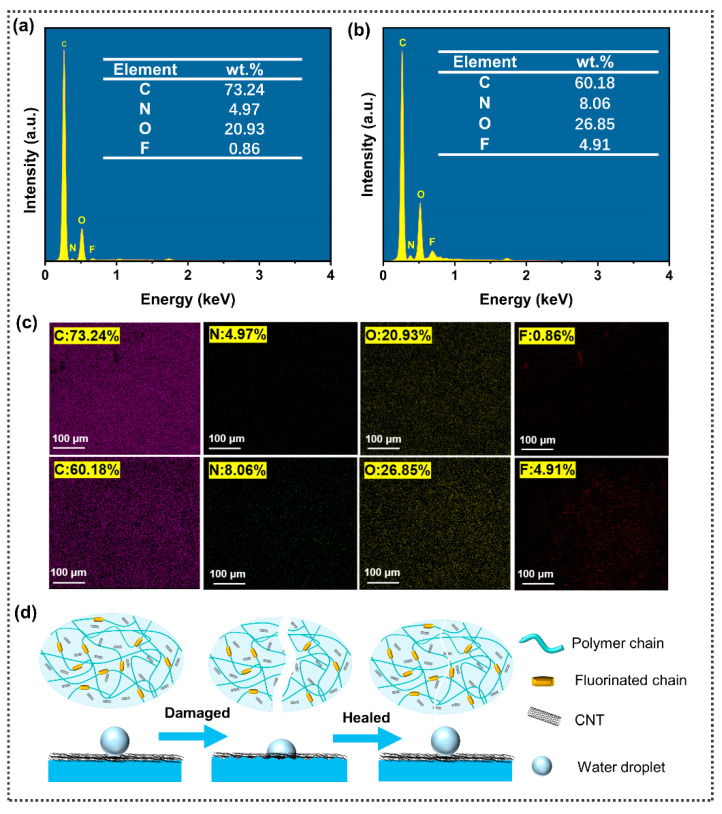
EDS spectrum of SFPUHE-HTF-CNTs: (**a**) before being damaged and (**b**) after self-healing. (**c**) Element distribution maps of SFPUHE-HTF-CNTs before being damaged and after self-healing. (**d**) Diagram of the hydrophobic self-healing mechanism of SFPUHE-HTF-CNTs.

To explore the self-healing mechanism of the hydrophobic property of the SFPUHE-HTF-CNTs, an EDS test was carried out on the SFPUHE-HTF-CNTs before and after the repair. It can be seen from Figure 6a,b that the contents of C, N, O and F of the original uncut sample were 73.24%, 4.97%, 20.93% and 0.86%, respectively. After the damage and the process of self-repair, the contents of C, N, O and F became 60.18%. 8.06%, 26.85% and 4.91%, respectively. Figure 6c, correspondingly, shows the element distribution maps of C, N, O and F before and after the self-repair. It can be seen from Figure 6c that the distribution of the elements on the surface of the sample was uniform. In addition, the content of the F element increased by 4.05% and the content of the O element increased by 5.89% compared with that before the self-repair. Based on the above results, the hydrophobic self-healing mechanism of the material was proposed as shown in Figure 6d. The good hydrophobic self-healing of the SFPUHE-HTF-CNTs originated from the migration of fluorinated polysiloxane chain segments to the surface. The glass transition temperature of HTF is much lower than 60 °C; therefore, the macromolecular chain segments can undergo thermal movement at this temperature. When the fluorine-containing groups on the surface of a coating are damaged and the hydrophobic property of the coating decreases, the fluorinated polysiloxane chain segments can migrate from the inside to the outside, so that the fluorine-containing groups with a low surface energy can be enriched on the surface of the coating again [45,46]. The hydrophobic property of the material can then be regained, thus, completing the hydrophobic self-healing process.

### 3.3. Anti-Corrosive Properties of SFPUHE-HTF-CNTs

**Figure 7:**
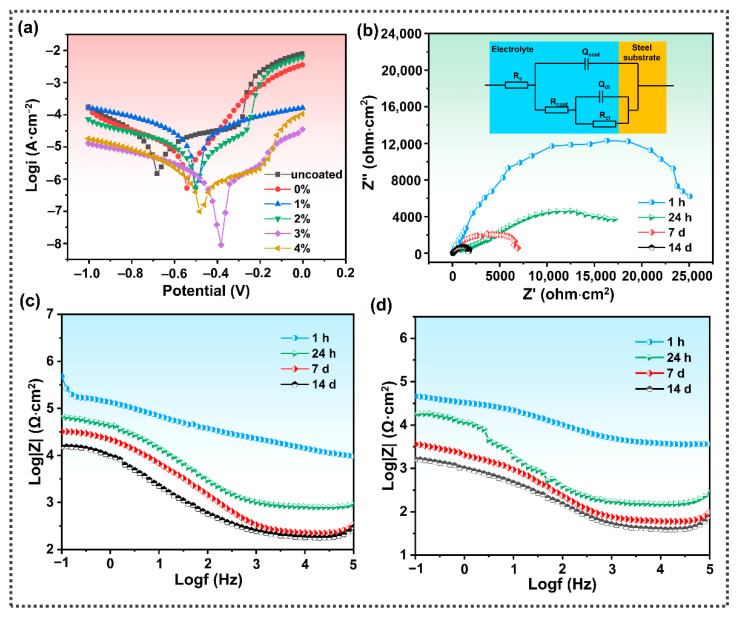
(**a**) Tafel polarization curves of the bare steel sheets and the coated steel sheets with different CNT contents immersed in a 3.5 wt% NaCl solution. (**b**) Nyquist plots of the SFPUHE-HTF-CNTs-3-coated steel sheets immersed in a 3.5 wt% NaCl solution for 1 h, 24 h, 7 d and 14 d; the inset is the equivalent circuit used to fit the EIS data. (**c**) Bode modulus plots of the SFPUHE-HTF-CNTs-3-coated steel sheets immersed in a 3.5 wt% NaCl solution for 1 h, 24 h, 7 d, 14 d. (**d**) Bode modulus maps of the repaired SFPUHE-HTF-CNTs-3-coated steel sheets immersed in a 3.5 wt% NaCl solution for 1 h, 24 h, 7 d, 14 d.

The skin-like bilayer structure design grants a coating the same self-healing function as a human epidermis and has shielding and anticorrosive effects. The anti-corrosion protective layer of a SFPUHE-HTF-CNT coating consists of CNTs, which can form zigzag barrier channels to shield corrosive media due to their large aspect ratio [47]. Here, the SFPUHE-HTF-CNTs were coated on steel sheets, and the effect of CNT contents on the corrosion resistance of the composite coating was explored by EIS in a 3.5 wt% NaCl solution. Figure 7a reveals the Tafel polarization curves of the bare and the coated steel sheet with different CNTs contents. The corrosion current density (I_corr_) and corrosion potential (E_corr_) of SFPUHE-HTF-CNTs with different CNT contents were obtained from the polarization curves, as shown in Table S1. As shown in Figure 7a and Table S1, the bare steel sheet had the highest corrosion current density and the lowest corrosion potential with an I_corr_ and E_corr_ of 1.47 × 10^−6^ A·cm^−2^ and −0.68 V, respectively. After coating with SFPUHE-HTF-CNT-0, the I_corr_ decreased to 5.24 × 10^−7^ A·cm^−2^ and the E_corr_ increased to −0.54 V. Past research has shown that a smaller I_corr_ and more positive E_corr_ represented lower corrosion trends and dynamic corrosion rates [48,49], indicating that a SFPUHE-HTF-CNT coating could effectively improve the corrosion resistance of the steel sheets. Additionally, as the content of the CNTs sprayed increased, the I_corr_ of the coated steel sheet showed a trend of decreasing and then increasing, while the E_corr_ was the opposite. The SFPUHE-HTF-CNTs-3-coated steel sheet had a relatively excellent anticorrosive effect, and its I_corr_ could reach 8.94 × 10^−9^ A·cm^−2^, reduced by two orders of magnitude relative to the SFPUHE-HTF-CNTs-0; the E_corr_ was −0.38 V and was shifted by 0.16 V in the positive direction relative to the SFPUHE-HTF-CNTs-0. It was thus clear that, due to the barrier and shielding effect of the CNTs layer, the bilayer SFPUHE-HTF-CNTs had a better anti-corrosion performance compared with the single-layer coating. However, the corrosion protection ability of the SFPUHE-HTF-CNTs decreased when the CNTs content was further increased to 4 wt.%, which might have been due to the tendency of agglomeration when the CNTs content was too high, thus, reducing the interaction with the polymer chain segments and making it easier for the corrosive media to penetrate into the steel substrate.

**Figure 8:**
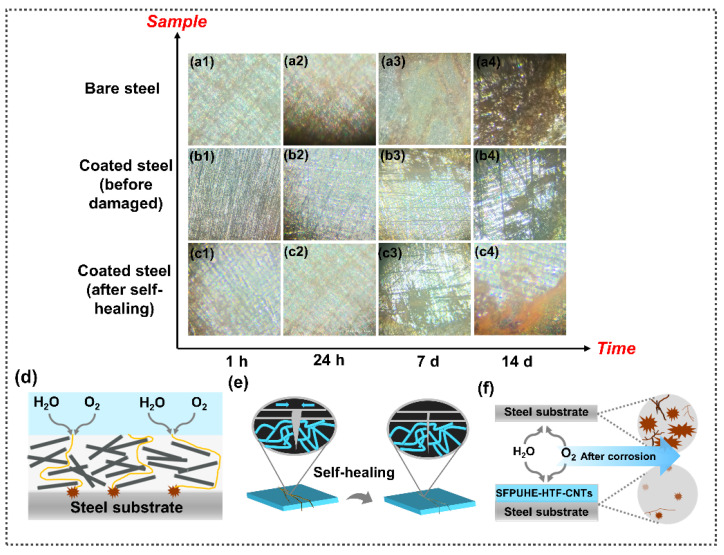
Micrographs (1000×) of surface corrosion of different samples in a 3.5 wt% NaCl solution for 1 h, 24 h, 7 d and 14 d: (**a1**–**a4**) bare steel sheets; and SFPUHE-HTF-CNTs-3-coated steel sheets (**b1**–**b4**) before and (**c1**–**c4**) after self-repair. (**d**) Schematic diagram of the zigzag barrier channels formed by CNTs in the outer layer of SFPUHE-HTF-CNTs. (**e**) Schematic diagram of the self-healing effect of the polyurethane inner layer of SFPUHE-HTF-CNTs. (**f**) Schematic diagram of the anti-corrosion effect of the skin-like bilayer structure coating of SFPUHE-HTF-CNTs.

Subsequently, the corrosion behavior of the bare steel sheet and the coated SFPU-HTF-CNTs-3 immersed in a 3.5 wt% NaCl solution for 1 h, 24 h, 7 d and 14 d, was further monitored by EIS. To clearly explain the corrosion mechanism, the equivalent circuit model was used to fit the obtained EIS data. The equivalent circuit of the sample is presented in the embedded illustration in Figure 7b. The parameters in the equivalent circuit represented the electrolyte resistance (R_s_), the polarization or charge transfer resistance of the substrate (R_ct_), the coating resistance (R_coat_), the capacitance to protect the coating (Q_coat_), and the double capacitance (Q_dl_) associated with the capacitance at the electrode/electrolyte interface [50]. The corresponding fitting results are summarized in Table 1. Generally, the R_ct_ is closely related to the corrosion process. It can be seen from Figure 7b that the coated SFPU-HTF-CNT-3 steel remained with a high diameter of the capacitor circuit, and the R_ct_ was up to 2.51 × 10^4^ Ω·cm^2;^ however, the R_ct_ after soaking for 24 h, 7 d and 14 d dropped to 1.71 × 10^4^ Ω·cm^2^, 6.91 × 10^3^ Ω·cm^2^ and 1.65 × 10^3^ Ω·cm^2^, respectively. This may have been caused by the gradual penetration of the corrosive medium into the coating with the extension of the soaking time; however, compared with the bare steel substrate, the coated SFPU-HTF-CNT-3 steel still exhibited a good corrosion resistance, which is subsequently confirmed in Figure 8(a1–c4). From Figure 7c, it can be seen that the coated steel sheet had a higher impedance modulus (|Z| = 4.79 × 10^5^ Ω·cm^2^, 1 h) after immersion in a NaCl solution. It was shown that the skin-like bilayer structure of the SFPUHE-HTF-CNTs had effective anticorrosive properties, which can be attributed to the good nano-size effect of CNTs prolonging the path of corrosive media (i.e., water, oxygen, etc.) into the steel substrate surface [51]. Figure 7d shows that the coated steel sheet still had a high impedance modulus (|Z| = 4.54 × 10^4^ Ω·cm^2^, 1 h) after self-healing, which proves the excellent anticorrosive self-healing properties of the composite coating. Similarly, the Tafel curve (Figure S3) confirmed this conclusion.

The surface corrosion of bare steel sheets and steel sheets coated with SFPUHE-HTF-CNTs-3 before and after self-repair were also observed by optical microscopy after immersion in a 3.5 wt% NaCl solution for 1 h, 24 h, 7 d, and 14 d. From Figure 8(a1–a4), it can be seen that with an extension of the soaking time, corrosive ions entered, so that the oxidation and corrosion on the steel sheet surface became increasingly serious. Figure 8(b1–b4) shows that when coated with SFPUHE-HTF-CNTs-3, thanks to the excellent barrier ability of the organic coating itself and the zigzag barrier channels formed after spraying CNTs on the surface, compared with the bare steel sheet, the corrosion on the surface of the metal steel reduced after coating for the same soaking time. From Figure 8(c1–c4), it can be seen that the SFPUHE-HTF-CNTs-3 layer after the damage and self-repair still had anti-corrosion performance after soaking. In summary, the SFPUHE-HTF-CNTs-3 coating significantly improved the corrosion resistance of the steel sheet and provided some anticorrosive self-healing capability. This process was due to the synergistic action of the inner and outer layers of the skin-like bilayer structure coating. The outer CNTs constitute a barrier channel to provide anticorrosion protection, and when the outer layer is damaged, the inner polyurethane layer can achieve self-healing of the overall coating that is driven by dynamic bonding. Meanwhile, the shielding effect of the outer layer provides a suitable environment for self-healing the inner layer [52]. Under the combined effect of the protection and self-healing functions, the anticorrosive self-healing process is completed (Figure 8d–f). The synergy between the inner and outer layers of the skin-like bilayer structure coating is of great significance to achieving intelligent self-healing corrosion protection.

### 3.4. Sensing Performance of SFPUHE-HTF-CNTs and Its Application in Real-Time Damage Monitoring

**Figure 9:**
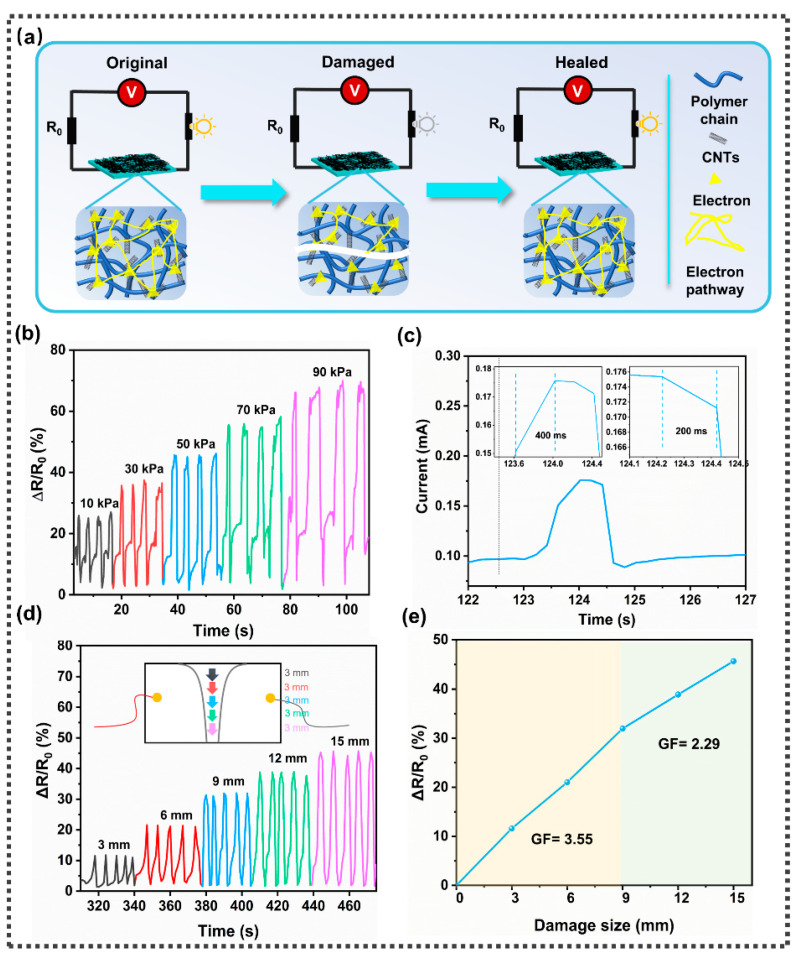
(**a**) Schematic diagram of the damage sensing mechanism. (**b**) Variations in relative resistance of SFPUHE-HTF-CNTs-3 at different pressures (i.e., 10 kPa, 30 kPa, 50 kPa, 70 kPa and 90 kPa). (**c**) Response time and recovery time of SFPUHE-HTF-CNTs-3. (**d**) Variations in relative resistance of SFPUHE-HTF-CNTs-3 at damage sizes (3 mm, 6 mm, 9 mm, 12 mm and 15 mm); the inset is a diagram of the coating subjected to different sizes of cuts. (**e**) Sensitivity curves of SFPUHE-HTF-CNTs-3 under different cuts of sizes.

Although self-repairing anticorrosion coatings can extend the service life by repairing damage, many damages are difficult to detect initially; therefore, the timely detection of damage and repair is essential to reduce maintenance costs and achieve long-lasting corrosion protection. Combining the outer layer of CNTs with the inner layer of an elastic matrix has become a commonly used strategy. When CNTs are sprayed onto the matrix, conductive pathways can be formed [53]. The damage can be responded to with a change in the resistance of the conductive network, which is conducive to the monitoring of defects (Figure 9a). Based on this design concept, SFPUHE-HTF-CNTs skin-like coatings were prepared, and thanks to the excellent conductivity of the outer CNTs, the SFPUHE-HTF-CNTs exhibited good electrical conductivity. Among them, the conductivity of SFPUHE-HTF-CNTs-3 could reach 112.92 S·m^−1^ after damage and self-repair (Table S2), and this good conductivity provided an excellent basis for the sensing performance of the composite coating.

Usually, damage is caused by external forces. Considering that SFPUHE-HTF-CNTs-3 has relatively excellent conductive self-healing properties, the pressure sensing performance of SFPUHE-HTF-CNTs-3 was examined by simulating pressure damage to the coating during an actual application by the pressure generated by weights. The normalized relative resistance change (ΔR/R_0_) was used to evaluate the responsiveness of the composite coating, where ΔR is the change in resistance in the pressure state and R_0_ is the original resistance in the unstressed state. Figure 9b shows that SFPUHE-HTF-CNTs-3 can respond to pressures from 10 kPa to 90 kPa, with a ΔR/R_0_ of 25.03%, 34.23%, 44.82%, 54.32%, and 65.90% when subjected to different levels of pressure, respectively, with a linear increasing trend and good repeatability. Figure 9c shows that the SFPUHE-HTF-CNTs-3 had a fast response time and recovery time of 400 ms and 200 ms, respectively, indicating that SFPUHE-HTF-CNTs-3 has the potential to be applied for the rapid detection of microcrack damage. Furthermore, to simulate different sizes of coating damage in practical applications, different sizes of cracks (i.e., 3 mm, 6 mm, 9 mm, 12 mm and 15 mm) were cut with a blade, and their electrical signal changes during the scratching process were examined. Figure 9d shows that SFPUHE-HTF-CNTs-3 can respond to different degrees of crack scratches with a linear increasing trend of ΔR/R_0_ as the scratch size increases. Figure 9e shows the sensitivity curve of SFPUHE-HTF-CNTs-3, which can be divided into two linear regions with different slopes, corresponding to two gauge factors (GFs), namely, 3.55 for the fracture range from 0 to 9 mm and 2.29 for the fracture range from 9 to 15 mm. The excellent sensing sensitivity indicates that SFPUHE-HTF-CNTs-3 can respond quickly to different levels of damage, which will be beneficial for its application in more complex environments.

**Figure 10:**
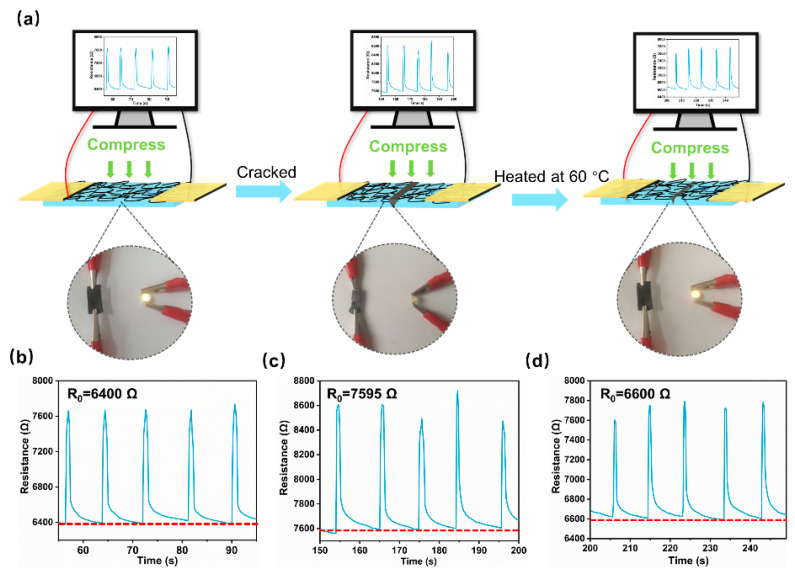
(**a**) Variations in resistance of SFPUHE-HTF-CNTs-3 at the states of being original, damaged, and healed ((**b**–**d**) are their enlarged views, respectively); the physical diagrams show the brightness of a bulb when the SFPUHE-HTF-CNTs were connected to the circuit. The resistive sensing signals of SFPUHE-HTF-CNTs-3: (**b**) original; (**c**) damaged; (**d**) healed.

A good sensing self-repair performance is a necessary prerequisite to determine whether a material can be continuously monitored for damages. To investigate the sensing self-repair ability of SFPUHE-HTF-CNTs-3, the changes in resistance signals before, during and after repair were examined under constant pressure (Figure 10a). From Figure 10b, it can be seen that the original SFPUHE-HTF-CNTs-3, due to the intact conductive path of CNTs when connected to the circuit, could make the small bulb glow with an initial resistance(R_0_) of 6400 Ω, and that the electrical signal remained relatively stable under constant pressure. In contrast, the R_0_ of the broken SFPUHE-HTF-CNTs-3 increased to 7595 Ω and the electrical signal was unstable due to the destruction of the conductive network (Figure 10c). When it was heated at 60 °C for 16 min, due to the conductive network that was restored, the bulb was lit again. The R_0_ was restored to 6600 Ω, and the electrical signal was relatively stable under constant pressure (Figure 10d). Usually, the conductive network will be rearranged after the self-healing material is damaged and self-repaired, which will affect the overall electrical properties of a material [54], but the skin-like SFPUHE-HTF-CNTs bilayer coating prepared here had an excellent recovery of the sensing signals. The good sensing self-healing ability facilitated the material continuously detecting internal cracks and external scratches, making its application and damage detection in complex environments possible.

## 4. Conclusions

In summary, a skin-like bilayer, self-healing SFPUHE-HTF-CNT smart anti-corrosion coating with disulfide bonds and fluorinated polysiloxane chain segments was reported. The SFPUHE-HTF-CNTs could self-repair cracks by heating at 60 °C for 16 min or irradiating under UV light at the energy of 200 for 48 h. The coating could be self-healed by thermal stimulation under mild conditions, which avoids the risk of material degradation due to a local concentrated heat. Additionally, the outer CNT corrosion protection layer possessed a superior barrier and excellent conductivity, with a low corrosion current density (8.94 × 10^−9^ A·cm^−2^), a positive corrosion potential (−0.38 V), and a high impedance modulus (|Z| = 4.79 × 10^5^ Ω·cm^2^). The impedance modulus still reached 4.54 × 10^4^ Ω·cm^2^ after self-healing, showing an excellent anti-corrosion self-repair performance. Morever, due to the formation of a conductive network by the CNTs, when the coating was damaged, the average distance between the CNTs changed and their conductivity changed; thus, the SFPUHE-HTF-CNTs also exhibited good damage-sensing performance and could achieve the real-time monitoring of damage with different pressures and sizes. This work not only provides a simple and effective avenue for preparing innovative, polyurethane-based, anti-corrosion coatings with real-time monitoring, but also combines it with advanced functions to expand the application to intelligent sensing, which is expected to be used in self-healing wearable-retractable electronics.

## Figures and Tables

**Table 1:** Fitting results of EIS data for bare steel and coated steel immersed in a 3.5 wt% NaCl solution for 1 h, 24 h, 7 d and 14 d.

Sample	R_s_ (Ω·cm^2^)	R_ct_ (Ω·cm^2^)	Q_dl_ (Ω^−1^·*s*^n^·cm^−2^)	R_coat_ (Ω·cm^2^)	Q_coat_ (Ω^−1^·*s*^n^·cm^−2^)
1 h	10.45	2.51 × 10^4^	5.45 × 10^−7^	4174	1.98 × 10^−6^
24 h	11.17	1.71 × 10^4^	7.55 × 10^−7^	3041	4.32 × 10^−6^
7 d	12.32	6.91 × 10^3^	1.01 × 10^−6^	2382	5.91 × 10^−6^
14 d	13.47	1.65 × 10^3^	1.74 × 10^−6^	1056	9.82 × 10^−6^

## Data Availability

The data presented in this study are available on request from the corresponding author.

## Supplementary Materials

The following supporting information can be downloaded at: https://www.mdpi.com/article/10.3390/nano13010124/s1, Figure S1: Raman spectra of SFPUHE-HTF; Figure S2a: Glass transition temperature of SFPU-HE0, SFPU-HE, SFPUHE-HTF and SFPUHE-HTF-CNTs determined by the isometric method; Figure S2b: Molecular structure formula of 1,4-BDO and HEDS; Figure S3: Tafel polarization curves of self-repaired coated SFPUHE-HTF-CNTs-3 steel sheets immersed in 3.5 wt% NaCl solution; Table S1: Corrosion current density and corrosion potential of SFPUHE-HTF-CNTs; Table S2: Conductivity of SFPUHE-HTF-CNTs before and after self-healing.
