# Supplementary material for: Self-Healing, Solvent-Free, Anti-Corrosion Coating Based on Skin-like Polyurethane/Carbon Nanotubes Composites with Real-Time Damage Monitoring
Source: Nanomaterials (Basel). 2022 Dec 26;13(1):124. doi: 10.3390/nano13010124 (PMC9823577; doi:10.3390/nano13010124)
Supplement: Supplementary file 1 [file nanomaterials-13-00124-s001.zip › nanomaterials-2018698-supplementary.pdf]

nanomaterials

Supplementary Materials

Self-Healing, Solvent-Free, Anti-Corrosion Coating Based on Skin-like Polyurethane/Carbon Nanotubes Composites with Real-Time Damage Monitoring

Hui Kong [1,2], Xiaomin Luo [1,2,*], Peng Zhang [1,2,*], Jianyan Feng [1,2], Pengni Li [3,4], Wenjie Hu [1,2], Xuechuan Wang [1,2] and Xinhua Liu [1,2]

1 College of Bioresources Chemical and Materials Engineering, Shaanxi University of Science & Technology, WeiYang District, Xi'an 710021, China
2 National Demonstration Center for Experimental Light Chemistry Engineering Education, Shaanxi University of Science & Technology, WeiYang District, Xi'an 710021, China
3 Tongxiang Affairs Center of Quality and Technical Supervision, Tongxiang 314599, China
4 National Wool Knitwear Quality Supervision Inspection Center (Zhe Jiang), Tongxiang 314599, China
* Correspondence: luoxiaomin@sust.edu.cn (X.L.); peter_zhang@sust.edu.cn (P.Z.); Tel.: +86-15809282916 (X.L.); +86-15616217730 (P.Z.)

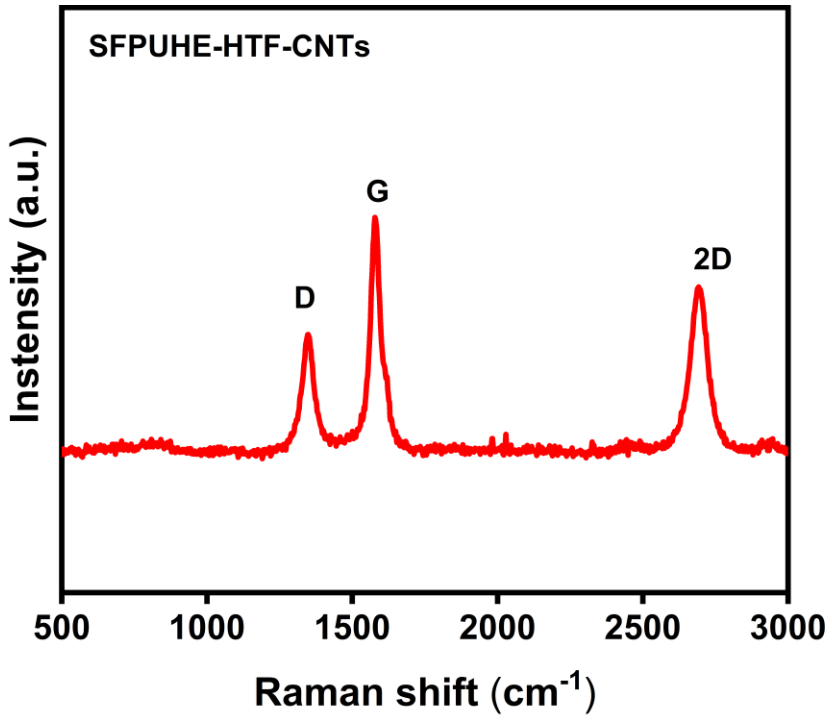

Figure S1. Raman spectrum of SFPUHE-HTF.

Figure S1 shows the Raman spectrum of SFPUHE-HTF-CNTs. Three characteristic Raman shifts appear in this figure, which correspond to the D peak (1353 cm^{-1}), G peak (1583 cm^{-1}) and 2 D peak (2686 cm^{-1}) of the CNTs. Notably, since the detection depth of Raman laser is only about 10 nm, so the SFPUHE-HTF-CNTs did not show the characteristic Raman shifts of sulfur-containing bonds; however, the appearance of characteristic Raman shifts associated with CNTs demonstrates the successful sprarying of CNTs on the surface of SFPUHE-HTF.

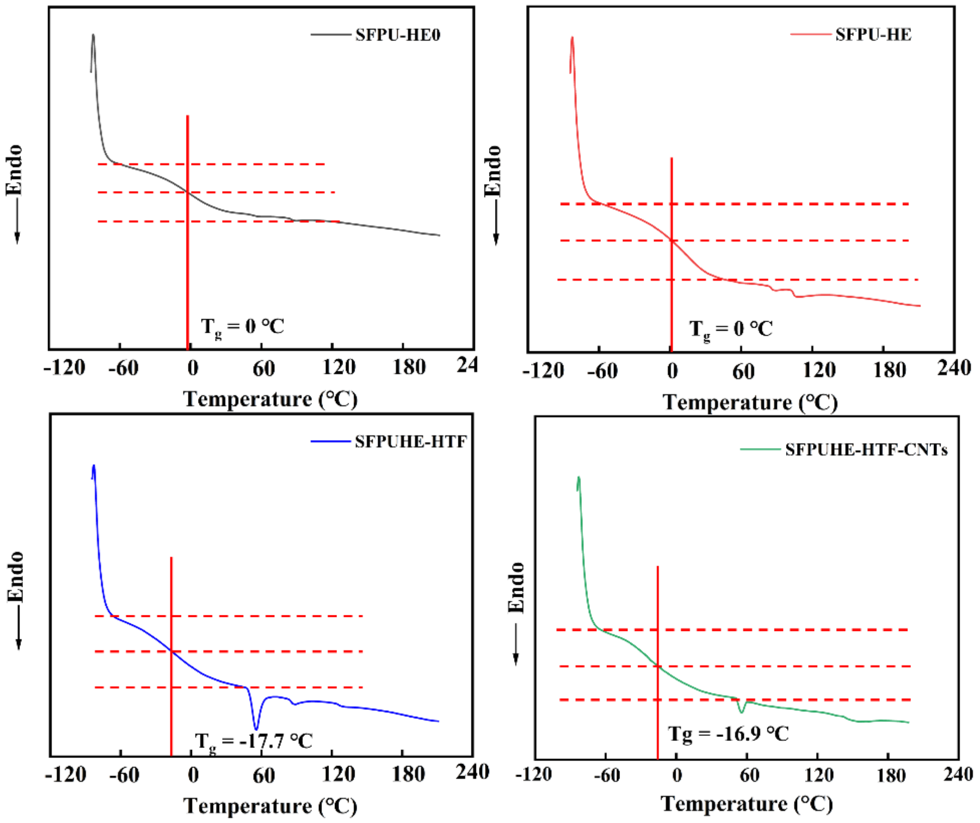

Figure S2a. Glass transition temperature of SFPU-HE0, SFPU-HE, SFPUHE-HTF and SFPUHE-HTF-CNTs determined by isometric method.

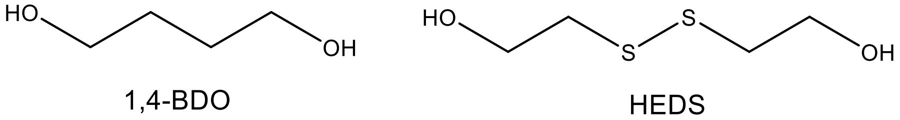

Figure S2b. Molecular structure formula of 1,4-BDO and HEDS.

Based on the test data of DSC, the "isometric method" was used to determine the Tg values of SFPU-HE0, SFPU-HE, SFPUHE-HTF and SFPUHE-HTF-CNTs, as displayed in **Figure S2a**. Although the components of the four soft segments are consistent, the components of the hard segments will also affect the T_g of the soft segments to a certain extent. As can be seen from **Figure S2a**, Tg values in the soft segment of the four samples are 0 °C, 0 °C, −17.7°C and −16.9 °C, respectively. SFPUHE-HTF possess the lowest Tg value in the soft segment, possibly because HTF contains Si-O bond and its internal rotation barrier is small, which improves the compliance of SFPUHE-HTF chain segment and thus shows low Tg value. SFPUHE-HTF-CNTs exhibit a similar Tg value to SFPUHE-HTF, which can be attributed to the similarity of their molecular chain structures. The slightly increase in Tg value of SFPUHE-HTF-CNTs is probably due to the formation of hydrogen bonds between CNTs and polyurethane molecules, which increases the proportion of the hard chain segments and leads to a decrease in molecular chain flexibility, thus result in a slightly higher glass transition temperature. As for SFPU-HE0 and SFPU-HE, they show similar Tg values. The difference between the two lies in the difference of chain extenders. The former uses BDO as chain extender, while the latter uses HEDS as chain extender. As can be seen from the chemical structure of HEDS and BDO **(Figure S2b)**, disulfide bond replaces the C-C bond, and there is little difference in the internal rotational potential energy between them. Therefore, it is also to be expected that they exhibit similar Tg values.

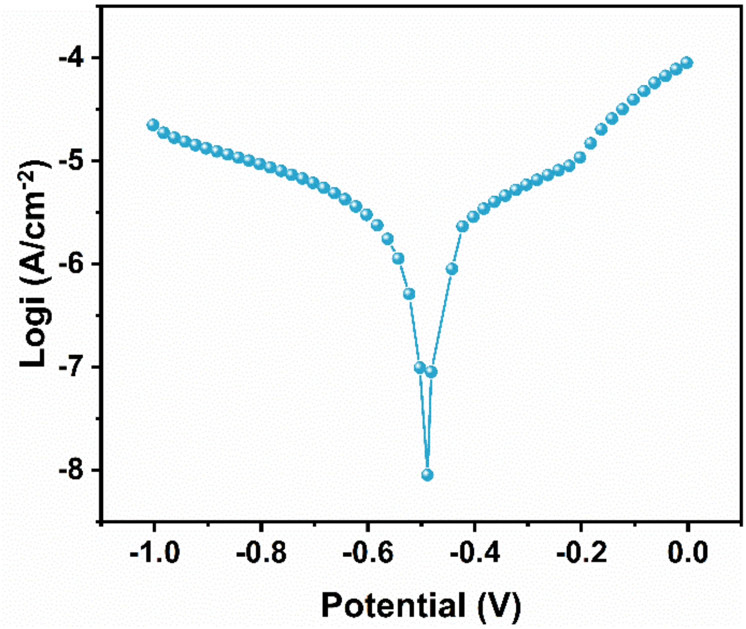

Figure S3. Tafel polarization curves of self-repaired coated SFPUHE-HTF-CNTs-3 steel sheets immersed in 3.5 wt% NaCl solution.

As depicted in **Figure S3**, compared with the original SFPUHE-HTF-CNTs-3, the damaged and self-repaired SFPUHE-HTF-CNTs-3 exhibited an increased corrosion current density of 9.12×10^{-9} A·cm^{-2} and a decreased corrosion potential of -0.49 V. Corrosion current density only increased by 2.01% and corrosion potential only decreased by 28.95%. Compared with the bare steel sheet ($I_{corr} = 1.47 \times 10^{-6}$ A·cm^{-2}, $E_{corr} = -0.68$ V), it still has a lower corrosion current density and a relatively positive corrosion potential, indicating that the self-repaired SFPUHE-HTF-CNTs coating still has good corrosion protection performance.

Table S1. Corrosion current density and corrosion potential of SFPUHE-HTF-CNTs.

**Samples**	**E_{corr} (V vs. SCE)**	**I_{corr} (A·cm^{-2})**
Uncoated	−0.68	1.47×10^{-6}
SFPUHE-HTF-CNTs-0	−0.54	5.24×10^{-7}
SFPUHE-HTF-CNTs-1	−0.50	5.50×10^{-7}
SFPUHE-HTF-CNTs-2	−0.50	5.41×10^{-7}
SFPUHE-HTF-CNTs-3	−0.38	8.94×10^{-9}
SFPUHE-HTF-CNTs-4	−0.48	9.78×10^{-8}

From **Table S1**, the I_{corr} and E_{corr} of the uncoated bare steel sheet are 1.47×10^{-6} A·cm^{-2} and −0.68 V, respectively. When coated with SFPUHE-HTF-CNTs composites, I_{corr} showed a trend of decreasing then increasing and E_{corr} presents the opposite. SFPUHE-HTF-CNTs-3 has the highest corrosion resistance, with corrosion current density up to 8.94×10^{-9} A·cm^{-2} and corrosion potential up to −0.38 V.

Table S2. Conductivity of SFPUHE-HTF-CNTs before and after self-healing.

**Samples**	**Conductivity (S·m^{-1})**	
	**original**	**healed**
SFPUHE-HTF-CNTs-0	—	—
SFPUHE-HTF-CNTs-1	1.46	1.34
SFPUHE-HTF-CNTs-2	43.51	11.20
SFPUHE-HTF-CNTs-3	180.74	112.92
SFPUHE-HTF-CNTs-4	270.27	106.38

The conductivity of SFPUHE-HTF-CNTs coating before and after self-healing is shown in **Table S2.** The conductivity of the coatings gradually increases with the increase of CNTs spraying amount, and has the highest conductivity up to 270.27 $S\cdot m^{-1}$ when the spraying amount is 4%. It is worth noting that the composite coating still has a high electrical conductivity after damage and self-healing. Taking the composite coating with 3% spraying content as an example, the conductivity after self-healing can still reach 112.92 $S\cdot m^{-1}$, which will be beneficial for the further application of the composite coating in damage detection.
